# Recent Insights into Cell Surface Heparan Sulphate Proteoglycans and Cancer

**DOI:** 10.12688/f1000research.8543.1

**Published:** 2016-06-29

**Authors:** John R Couchman, Hinke Multhaupt, Ralph D. Sanderson

**Affiliations:** 1 Department of Biomedical Sciences and Biotech Research & Innovation Center, University of Copenhagen, Copenhagen, Denmark; 2 Department of Pathology and University of Alabama at Birmingham Comprehensive Cancer Center, University of Alabama at Birmingham, Birmingham, AL, USA

**Keywords:** Heparan sulphate, heparanase, exosomes, Cell surface proteoglycans

## Abstract

A small group of cell surface receptors are proteoglycans, possessing a core protein with one or more covalently attached glycosaminoglycan chains. They are virtually ubiquitous and their chains are major sites at which protein ligands of many types interact. These proteoglycans can signal and regulate important cell processes, such as adhesion, migration, proliferation, and differentiation. Since many protein ligands, such as growth factors, morphogens, and cytokines, are also implicated in tumour progression, it is increasingly apparent that cell surface proteoglycans impact tumour cell behaviour. Here, we review some recent advances, emphasising that many tumour-related functions of proteoglycans are revealed only after their modification in processes subsequent to synthesis and export to the cell surface. These include enzymes that modify heparan sulphate structure, recycling of whole or fragmented proteoglycans into exosomes that can be paracrine effectors or biomarkers, and lateral interactions between some proteoglycans and calcium channels that impact the actin cytoskeleton.

## Introduction

Proteoglycans are present in all cellular and tissue compartments. Moreover, in mammals they are expressed by virtually all cells. By definition, proteoglycans consist of a core protein to which one or more glycosaminoglycan chains are covalently attached. While the number of proteoglycan core proteins in the mammalian genome is not large, their form and functions are highly variable. Aggrecan, a major constituent of cartilage matrix, for example, may have >100 chondroitin sulphate chains, which are key to its function in the maintenance of a hydrated, compression-resisting matrix ^1, 2^. Decorin, on the other hand, with roles in collagen fibril formation and regulation of innate immunity, has only one chondroitin or dermatan sulphate chain ^3^. Not surprisingly, since proteoglycans can be intracellular, cell surface, or extracellular matrix components, they are increasingly studied in the context of tumour growth, the tumour and stem cell niche, and invasion, metastasis, and tumour-host interactions ^4– 9^.

On the surfaces of most mammalian cells are representatives of two major families of heparan sulphate proteoglycans (HSPGs), the glypicans and syndecans ^5, 10– 12^. The former are linked to the membrane through a glycosylphosphatidylinositol anchor, while the syndecans are transmembrane, with a highly conserved short cytoplasmic domain. Usually the core proteins carry two to five heparan sulphate chains, but syndecans may sometimes also, or alternately, carry chondroitin or dermatan sulphate chains ^5^. The synthesis of heparan sulphate chains is a complex Golgi apparatus-localised process; while all of the transferases and other modifying enzymes involved in their synthesis are known, their regulation is not ^13^. The importance of heparan sulphate synthesis lies in the fact that this glycosaminoglycan has an ability to interact with a wide array of binding partners that include cytokines, chemokines, growth factors, extracellular matrix macromolecules, enzymes, and lipoproteins ^14, 15^. Heparan sulphate chains have regions of high modification (i.e. high levels of sulphation) interspersed with regions of low, or no, sulphation ^15^. This most complex of all post-translational modifications is under scrutiny, since most protein binding partners of heparan sulphate engage with highly sulphated domains ^14, 16^, so the control of its synthesis and how this may change with transformation are important issues. Moreover, mature heparan sulphate chains can be further modified by a single mammalian heparanase enzyme and by two sulphatases that selectively remove the sulphates of some glucosamine residues ^17– 19^. Heparan sulphate editing is now a topic of great interest in tumour biology and some recent developments are summarised below.

For many years, it was assumed that cell surface HSPGs had few independent functions but were mostly acting *in cis* as co-receptors with other receptors, e.g., tyrosine kinase growth factor receptors and integrins ^5, 11, 12, 20^. The notion was that the heparan sulphate chains provided binding sites for ligands that could then be concentrated for high-affinity receptor binding and subsequent signalling. It now seems clear that there are more intricate interactions at the cell surface that involve independent roles for the cell surface HSPGs. Some of the latest insights into cell surface HSPG functions with relevance to tumour biology are briefly reviewed here. Recent information on the roles of other classes of extracellular matrix proteoglycans in cancer can be found elsewhere ^3, 4, 7, 9, 21^.

## Heparan sulphate editing: regulatory events in tumour progression

There is abundant evidence that heparan sulphates, owing to their diversity in structure and location, play important roles in regulating the growth and progression of cancer. Much of this regulation occurs *via* the ability of heparan sulphate to fine-tune molecular interactions that regulate cell behaviour ^22^. Over the last decade, it has become increasingly apparent that enzymes can edit heparan sulphate structure, thereby precisely modulating its function and regulating cell behaviour. These enzymes include the endoglucuronidase heparanase, which cleaves and shortens heparan sulphate chains of proteoglycans that as a consequence possess new non-reducing termini, and the extracellular sulphatases Sulf-1 and -2 that selectively remove 6- *O* sulphates. Both of these enzyme activities are proving to be powerful regulators of tumour behaviour.

Heparanase is associated with aggressive tumour behaviour including enhanced growth, angiogenesis, and metastasis. Although a number of studies in many tumour types have supported these conclusions, a unifying mechanistic explanation of precisely how heparanase promotes angiogenesis and metastasis was lacking until recently. In a paper just published in Oncogenesis, Jung *et al*. demonstrate that heparanase-mediated trimming of syndecan-1 heparan sulphate chains and upregulation of matrix metalloproteinase-9 (MMP-9) expression results in enhanced shedding of syndecan-1 from the cell surface. Shedding exposes a juxtamembrane site on the syndecan-1 core protein that binds to both very late antigen-4 (VLA-4 [integrin α4β1]) and vascular endothelial growth factor receptor-2 (VEGFR2). This coupling of VLA-4 to VEGFR2 activates the latter, thereby initiating downstream signalling that displaces the cytoskeletal adaptor protein paxillin from VLA-4, in turn facilitating the activation of Rac GTPase and polarised cell migration ^23^. This mechanism is in play on both endothelial cells and tumour cells and demonstrates how heparanase, in concert with syndecan-1, drives angiogenesis, tumour cell invasion, and subsequent metastasis.

Evidence is also emerging that heparanase plays a key role in promoting chemoresistance. In breast cancer cell lines expressing a high level of heparanase, inhibition of the enzyme sensitised the cells to killing by lapatinib ^24^. Elevated heparanase expression by myeloma cells enhances their resistance to both bortezomib and melphalan and this resistance is reversed *in vivo* when mice are treated with the heparanase inhibitor Roneparstat ^25^. Furthermore, heparanase was shown to be present at a high level on tumour cells that survive extensive chemotherapy in myeloma patients, lending further support to the notion that heparanase promotes resistance to therapy ^25^. Together, these findings raise the exciting possibility that the efficacy of anti-cancer drugs may be enhanced when combined with the use of heparanase inhibitors. This is of particular interest, as there are currently four anti-heparanase drugs in clinical trials in cancer patients ^19^. These drugs are all heparin mimetics that are thought to inhibit heparanase activity by blocking the enzyme’s active site. However, recent solving of the crystal structure of heparanase provides an opportunity for the discovery of small molecule inhibitors of enzyme activity that should exhibit improved specificity over the heparin mimetics ^26^. Heparanase-neutralising antibodies have also recently shown promise in attenuating the growth and metastasis of lymphoma and myeloma tumours in mice ^27^.

While heparanase may have important roles in supporting tumour angiogenesis, it is important to recognise that it is not the only mechanism. Many angiogenesis-promoting growth factors, such as VEGF, fibroblast growth factors (FGFs), cytokines, and chemokines, have high affinity for heparan sulphate. It is therefore likely that vascular remodelling is a consequence of multiple interactions involving cell surface HSPGs ^14, 28– 30^.

**Figure 1.**
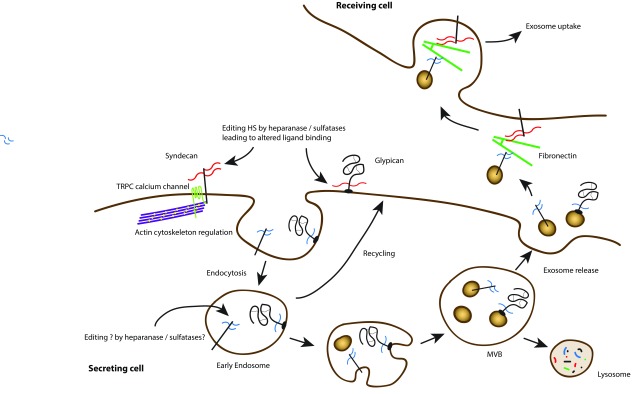
Cell surface proteoglycans regulate cell communication Cell surface heparan sulphate proteoglycans can interact with multiple ligands through their glycosaminoglycan chains. In addition, they can be modified by heparanase and sulphatases, leading to altered ligand binding. Endocytosis, trafficking, and processing can lead to the release of exosomes bearing modified proteoglycans. These can interact with fibronectin in the extracellular environment and ultimately be bound and internalised by recipient cells. This signalling at a distance may be important in the regulation of tumour cell behaviour.

Although it is generally agreed that the function of Sulf-1 and -2 is to selectively remove 6- *O* sulphates from heparan sulphate chains, the impact of these two extracellular sulphatases on tumour growth and progression remains controversial. By altering the composition of heparan sulphates, the Sulfs regulate the signalling capacity of heparin-binding growth factors such as Wnts, FGF, EGF, and VEGF, among others ^19^. Predictably, this has important consequences for tumour behaviour. What is surprising is that despite their seemingly identical function, there are data to support the conclusion that Sulf-1 suppresses tumour growth while Sulf-2 promotes tumour growth ^31, 32^. However, such a generalisation appears to be misleading because there is evidence that in some instances Sulf-1 promotes, while Sulf-2 inhibits, tumour growth. Together, these findings strongly suggest that there are factors beyond the catalytic activity of the Sulfs that determine their ultimate impact on tumour behaviour ^31, 33, 34^ ( Figure 1). Such factors may be related to spatial or temporal expression of the Sulfs, variations in their specificity for the heparan sulphate substrate, or differing abilities of the Sulfs to diffuse through the tumour microenvironment. Moreover, there is evidence for non-catalytic properties of Sulfs that lead to alterations in heparan sulphate synthesis through changes in sulphotransferase expression ^33^ or upregulation of glypican-3 core protein, which is relevant to hepatocellular carcinoma ^34^.

## Signalling at a distance through exosomes

In 2012, the first of several papers was published suggesting that syndecans were cell surface receptors important in exosome formation ^35^. For this, the most C-terminal region of the syndecan cytoplasmic domain interacting with PDZ domain proteins was required. The cytoplasmic scaffolding protein syntenin (also known as melanoma differentiation-associated gene 9; MDA-9) binds to all syndecans through one of its two PDZ domains ^36, 37^, and this was shown to be important for the endosomal and trafficking events that lead to exosome formation ^38^. The other PDZ domain of syntenin had high affinity for the membrane phospholipid phosphatidylinositol 4,5-bisphosphate (PtdIns4,5P _2_). Syntenin also interacts through its C-terminal domain with Bro1/ALG-2-interacting protein (ALIX ^39^), a central player in exosome formation. In turn, ALIX links to a multiprotein endosomal sorting complex required for transport (ESCRT), with additional roles for the GTPase Arf6 and phospholipase D2 ^40^. Exosomes are now recognised as important signalling vesicles, containing a number of proteins, lipids, and even nucleic acids such as RNAs and miRNAs. They are produced by most cells, including tumour cells, and interest in them from the tumour perspective focuses on whether they can be detectable biomarkers in fluids and their potential roles in regulating the tumour environment ( Figure 1). Moreover, syntenin (MDA-9) was first identified in the context of melanoma but is upregulated in many tumours where experiments have shown that it supports cell migration or invasion ^37, 41^. It has many binding partners beyond syndecans, including the tetraspanin CD63, an exosome marker ^42^, but what controls the selectivity of syntenin to interact with many different cell surface molecules is currently unclear. However, it has been suggested that this protein is a potential tumour target ^43^.

Interestingly, similar to their roles in regulating tumour angiogenesis and metastasis, heparanase and syndecans also work together in regulating exosome secretion by tumour cells. Enhanced heparanase expression in tumour cells stimulates exosome biogenesis, alters exosome protein composition, and enhances the ability of exosomes to promote tumour cell spreading and endothelial cell migration ^44^. In this instance, heparan sulphate chains of syndecans are essential for exosome formation within endosomal compartments, and trimming of heparan sulphate by heparanase activates the formation of an endosomal complex containing syndecan coupled to syntenin and ALIX ^35, 45^. This complex promotes endosomal membrane budding and drives exosome biogenesis. Following their secretion, exosomes exert their biological activity by docking with recipient cells and delivering cargo that can alter recipient cell behaviour. In this context, the heparan sulphate present on syndecan, which remains on the exosome surface following the biogenesis process, can interact with fibronectin *via* its Hep-II heparin-binding domain ^46^. The fibronectin-coated exosomes subsequently dock by binding to the heparan sulphate of proteoglycans present on the recipient cell surface. At least in some cases, the heparan sulphate present on recipient cells can also act as an internalising receptor, thus facilitating the uptake of exosomes and subsequent delivery of exosome cargo within the cell ^47^ ( Figure 1).

Syndecans are not the only proteoglycans with potential importance to exosomes. In 2015, a very interesting report documented that circulating exosomes containing glypican-1 could potentially identify patients with pancreatic ductal adenocarcinoma, even at early stages of tumour development ^48^. Whether the heparan sulphate chains were present and carrying important growth factors, cytokines, or chemokines remains speculative, but once more the connection between cell surface HSPGs and cancer is apparent.

## Syndecans, cytoskeleton, adhesion, and migration

The four mammalian syndecans all interact with the actin cytoskeleton ^5^. Much research has been devoted to understanding this relationship, and many reports have provided evidence that they contribute to microfilament organisation in adhesion and migration. Perhaps the best example in this regard is syndecan-4. It promotes the assembly of focal adhesions, junctions that form in response to cell adhesion to the extracellular matrix. They are integrin-dependent organelles, but the mechanism by which syndecan influences the process has taken many years to unravel. Key to syndecan-4’s role are interactions with both the actin-associated protein α-actinin ^49– 51^ and protein kinase Cα, through which there are multiple potential pathways involving Rho family GTPases to the cytoskeleton ^52, 53^. The roles of RhoA, Rac, and cdc42 are well known in this regard ^54, 55^. Analysis of fibroblasts derived from syndecan-4 null mice show clear differences in microfilament organisation, with much reduced focal adhesions and stress fibres ^51, 56, 57^, for which RhoGTPase activities seem not to provide the whole explanation. Recent analysis has now shown that this altered adhesion phenotype of S4KO cells relates to calcium channels of the TRPC (transient receptor potential canonical) family. Indeed, elimination of the TRPC7 channel (itself a focal adhesion component) reverts the S4KO cells to wild-type in terms of adhesion, cytoskeleton, and junction formation ^58^. This was accompanied by reductions in cytosolic calcium that were shown to be increased in the null cells compared to matching wild-type cells. Further work with epithelial cells and, moreover, genetic experiments with *Caenorhabditis elegans* (which possesses a single syndecan) show that this regulation of TRPC type channels by syndecans may be a highly conserved and important role for this proteoglycan family ^58^.

The work with syndecans and channels has so far not embraced tumour cells. Since calcium is a potent regulator of the actin cytoskeleton, it may now be attractive to re-examine some of the previous observations on HSPGs and tumour cells. The literature is replete with studies showing that syndecans are often mis-expressed in solid tumours and in some cases relate to prognosis ^59– 62^. A good example is breast cancer, where high levels of syndecan-1 expression, particularly in the tumour stroma, are an indicator of poor prognosis ^63, 64^. In other studies, syndecan-2 upregulation has been shown to alter the adhesion and invasiveness of MDA-MB231 breast carcinoma cells and colon carcinoma cells ^65, 66^. The difficulty with many studies is understanding whether syndecan expression merely correlates with or is functionally related to tumour progression. In some cases, however, the situation is clearer. A wealth of evidence now suggests that syndecan-1 expression in myeloma is related directly to disease severity and progression ^67, 68^. Moreover, it is not only syndecans that may influence tumour progression. Evidence has accumulated rapidly over the past few years showing a relationship between glypican-3 expression and the progression of hepatocellular carcinoma ^69– 71^. This HSPG is expressed in foetal liver, but levels subside in postnatal life ^72, 73^. However, in a large majority of cases, glypican-3 is re-expressed in hepatocellular carcinoma ^72, 74^. The excitement about this HSPG revolves around the possibility that it may serve as a prognostic marker, but also a target for immunotherapy ^71^. Early clinical trials have been reported, but clearly there is a long way to go. On a molecular level, it has been suggested that glypican-3 can bind both Wnt and Frizzled, the signalling receptor for Wnts, through its heparan sulphate chains ^70, 71^. However, the situation is complex, since glypican-3 in normal tissue may be a growth inhibitor. Rare core protein mutations giving rise to the Simpson-Golabi-Behmel syndrome are characterised by overgrowth and many dysmorphisms in patients and a corresponding murine model ^75^. In hepatocellular carcinoma, however, there is also upregulation of Sulf-2. It now appears that selective removal of 6- *O*-sulphate residues from the glypican’s heparan sulphate chains leads to Wnt activation, possibly through its enhanced mobility, leading to Frizzled binding and signalling ^76^. It is also possible that the heparan sulphate chains may bind hepatocyte growth factor and members of the FGF family ^77, 78^.

## Conclusions

Recent developments have highlighted that both the heparan sulphate chains and the core proteins of cell surface HSPGs are highly and functionally relevant to tumour progression. Moreover, the increasingly recognised importance of the tumour cell niche ^79, 80^, which is rich in proteoglycans, and the emerging roles of proteoglycans in stem cell differentiation ^6, 81^ are areas for future development. Moreover, it is not only HSPGs that present as targets in tumours. The chondroitin sulphate proteoglycan 4 (also known as NG2) is recognised as a cell surface marker of pericytes in the vasculature but is also present more widely, for example on neuronal and oligodendrocyte precursors ^82^. It is also an emerging target for immunotherapy in a variety of tumour types, including melanoma, triple negative breast cancer, glioblastoma, mesothelioma, and sarcomas ^83, 84^.

The potential for cell surface proteoglycans to be targets for intervention are complicated by their multiple roles and ubiquity. It is perhaps likely that tumour cells, stromal/other host tissue, and the immune system utilise these proteoglycans and their downstream signalling in specific ways to regulate behaviour. Targeting will require detailed understanding, and therefore we can predict that new insights into the functions of proteoglycans will impact tumour biology for many years to come.

## References

[1] Aspberg A: The different roles of aggrecan interaction domains. *J Histochem Cytochem.* 2012;60(12):987–96. 10.1369/0022155412464376 23019016 PMC3527881

[2] Heinegård D Saxne T: The role of the cartilage matrix in osteoarthritis. *Nat Rev Rheumatol.* 2011;7(1):50–6. 10.1038/nrrheum.2010.198 21119607

[3] Neill T Schaefer L Iozzo RV: Decoding the Matrix: Instructive Roles of Proteoglycan Receptors. *Biochemistry.* 2015;54(30):4583–98. 10.1021/acs.biochem.5b00653 26177309 PMC4859759

[4] Iozzo RV Sanderson RD: Proteoglycans in cancer biology, tumour microenvironment and angiogenesis. *J Cell Mol Med.* 2011;15(5):1013–31. 10.1111/j.1582-4934.2010.01236.x 21155971 PMC3633488

[5] Couchman JR: Transmembrane signaling proteoglycans. *Annu Rev Cell Dev Biol.* 2010;26:89–114. 10.1146/annurev-cellbio-100109-104126 20565253

[6] Pickford CE Holley RJ Rushton G: Specific glycosaminoglycans modulate neural specification of mouse embryonic stem cells. *Stem Cells.* 2011;29(4):629–40. 10.1002/stem.610 21308866

[7] Schaefer L Iozzo RV: Small leucine-rich proteoglycans, at the crossroad of cancer growth and inflammation. *Curr Opin Genet Dev.* 2012;22(1):56–7. 10.1016/j.gde.2011.12.002 22326829

[8] Multhaupt HA Leitinger B Gullberg D: Extracellular matrix component signaling in cancer. *Adv Drug Deliv Rev.* 2016;97:28–40. 10.1016/j.addr.2015.10.013 26519775

[9] Theocharis AD Gialeli C Bouris P: Cell-matrix interactions: focus on proteoglycan-proteinase interplay and pharmacological targeting in cancer. *FEBS J.* 2014;281(22):5023–42. 10.1111/febs.12927 25333340 PMC5036392

[10] Filmus J Capurro M Rast J: Glypicans. *Genome Biol.* 2008;9(5):224. 10.1186/gb-2008-9-5-224 18505598 PMC2441458

[11] Morgan MR Humphries MJ Bass MD: Synergistic control of cell adhesion by integrins and syndecans. *Nat Rev Mol Cell Biol.* 2007;8(12):957–69. 10.1038/nrm2289 17971838 PMC3329926

[12] Couchman JR Gopal S Lim HC: Syndecans: from peripheral coreceptors to mainstream regulators of cell behaviour. *Int J Exp Pathol.* 2015;96(1):1–10. 10.1111/iep.12112 25546317 PMC4352346

[13] Multhaupt HA Couchman JR: Heparan sulfate biosynthesis: methods for investigation of the heparanosome. *J Histochem Cytochem.* 2012;60(12):908–15. 10.1369/0022155412460056 22899865 PMC3527879

[14] Xu D Esko JD: Demystifying heparan sulfate-protein interactions. *Annu Rev Biochem.* 2014;83:129–57. 10.1146/annurev-biochem-060713-035314 24606135 PMC7851832

[15] Gallagher J: Fell-Muir Lecture: Heparan sulphate and the art of cell regulation: a polymer chain conducts the protein orchestra. *Int J Exp Pathol.* 2015;96(4):203–31. 10.1111/iep.12135 26173450 PMC4561558

[16] Lindahl U Li JP: Interactions between heparan sulfate and proteins-design and functional implications. *Int Rev Cell Mol Biol.* 2009;276:105–59. 10.1016/S1937-6448(09)76003-4 19584012

[17] Lamanna WC Kalus I Padva M: The heparanome--the enigma of encoding and decoding heparan sulfate sulfation. *J Biotechnol.* 2007;129(2):290–307. 10.1016/j.jbiotec.2007.01.022 17337080

[18] Rosen SD Lemjabbar-Alaoui H: Sulf-2: an extracellular modulator of cell signaling and a cancer target candidate. *Expert Opin Ther Targets.* 2010;14(9):935–49. 10.1517/14728222.2010.504718 20629619 PMC3126665

[19] Hammond E Khurana A Shridhar V: The Role of Heparanase and Sulfatases in the Modification of Heparan Sulfate Proteoglycans within the Tumor Microenvironment and Opportunities for Novel Cancer Therapeutics. *Front Oncol.* 2014;4:195. 10.3389/fonc.2014.00195 25105093 PMC4109498

[20] Bernfield M Götte M Park PW: Functions of cell surface heparan sulfate proteoglycans. *Annu Rev Biochem.* 1999;68:729–77. 10.1146/annurev.biochem.68.1.729 10872465

[21] Poluzzi C Iozzo RV Schaefer L: Endostatin and endorepellin: A common route of action for similar angiostatic cancer avengers. *Adv Drug Deliv Rev.* 2016;97:156–73. 10.1016/j.addr.2015.10.012 26518982 PMC4753091

[22] Bishop JR Schuksz M Esko JD: Heparan sulphate proteoglycans fine-tune mammalian physiology. *Nature.* 2007;446(7139):1030–7. 10.1038/nature05817 17460664

[23] Jung O Trapp-Stamborski V Purushothaman A: Heparanase-induced shedding of syndecan-1/CD138 in myeloma and endothelial cells activates VEGFR2 and an invasive phenotype: prevention by novel synstatins. *Oncogenesis.* 2016;5:e202. 10.1038/oncsis.2016.5 26926788 PMC5154350

[24] Zhang L Ngo JA Wetzel MD: Heparanase mediates a novel mechanism in lapatinib-resistant brain metastatic breast cancer. *Neoplasia.* 2015;17(1):101–13. 10.1016/j.neo.2014.11.007 25622903 PMC4309682

[25] Ramani VC Zhan F He J: Targeting heparanase overcomes chemoresistance and diminishes relapse in myeloma. *Oncotarget.* 2016;7(2):1598–607. 10.18632/oncotarget.6408 26624982 PMC4811483

[26] Wu L Viola CM Brzozowski AM: Structural characterization of human heparanase reveals insights into substrate recognition. *Nat Struct Mol Biol.* 2015;22(12):1016–22. 10.1038/nsmb.3136 26575439 PMC5008439

[27] Weissmann M Arvatz G Horowitz N: Heparanase-neutralizing antibodies attenuate lymphoma tumor growth and metastasis. *Proc Natl Acad Sci U S A.* 2016;113(3):704–9. 10.1073/pnas.1519453113 26729870 PMC4725485

[28] Jakobsson L Kreuger J Holmborn K: Heparan sulfate in *trans* potentiates VEGFR-mediated angiogenesis. *Dev Cell.* 2006;10(5):625–34. 10.1016/j.devcel.2006.03.009 16678777

[29] Matsuo I Kimura-Yoshida C: Extracellular modulation of Fibroblast Growth Factor signaling through heparan sulfate proteoglycans in mammalian development. *Curr Opin Genet Dev.* 2013;23(4):399–407. 10.1016/j.gde.2013.02.004 23465883

[30] Peysselon F Ricard-Blum S: Heparin-protein interactions: from affinity and kinetics to biological roles. Application to an interaction network regulating angiogenesis. *Matrix Biol.* 2014;35:73–81. 10.1016/j.matbio.2013.11.001 24246365

[31] Vivès RR Seffouh A Lortat-Jacob H: Post-Synthetic Regulation of HS Structure: The Yin and Yang of the Sulfs in Cancer. *Front Oncol.* 2014;3:331. 10.3389/fonc.2013.00331 24459635 PMC3890690

[32] Yang JD Sun Z Hu C: Sulfatase 1 and sulfatase 2 in hepatocellular carcinoma: associated signaling pathways, tumor phenotypes, and survival. *Genes Chromosomes Cancer.* 2011;50(2):122–35. 10.1002/gcc.20838 21104785 PMC3253341

[33] Lamanna WC Frese MA Balleininger M: Sulf loss influences *N-, 2-O-,* and *6-O-*sulfation of multiple heparan sulfate proteoglycans and modulates fibroblast growth factor signaling. *J Biol Chem.* 2008;283(41):27724–35. 10.1074/jbc.M802130200 18687675

[34] Lai JP Sandhu DS Yu C: Sulfatase 2 up-regulates glypican 3, promotes fibroblast growth factor signaling, and decreases survival in hepatocellular carcinoma. *Hepatology.* 2008;47(4):1211–22. 10.1002/hep.22202 18318435 PMC2536494

[35] Baietti MF Zhang Z Mortier E: Syndecan-syntenin-ALIX regulates the biogenesis of exosomes. *Nat Cell Biol.* 2012;14(7):677–85. 10.1038/ncb2502 22660413

[36] Grootjans JJ Zimmermann P Reekmans G: Syntenin, a PDZ protein that binds syndecan cytoplasmic domains. *Proc Natl Acad Sci U S A.* 1997;94(25):13683–8. 10.1073/pnas.94.25.13683 9391086 PMC28366

[37] Das SK Bhutia SK Kegelman TP: MDA-9/syntenin: a positive gatekeeper of melanoma metastasis. *Front Biosci (Landmark Ed).* 2012;17:1–15. 10.2741/3911 22201728

[38] Ghossoub R Lembo F Rubio A: Syntenin-ALIX exosome biogenesis and budding into multivesicular bodies are controlled by ARF6 and PLD2. *Nat Commun.* 2014;5: 3477. 10.1038/ncomms4477 24637612

[39] Théry C Boussac M Véron P: Proteomic analysis of dendritic cell-derived exosomes: a secreted subcellular compartment distinct from apoptotic vesicles. *J Immunol.* 2001;166(12):7309–18. 10.4049/jimmunol.166.12.7309 11390481

[40] Friand V David G Zimmermann P: Syntenin and syndecan in the biogenesis of exosomes. *Biol Cell.* 2015;107(10):331–41. 10.1111/boc.201500010 26032692

[41] Kashyap R Roucourt B Lembo F: Syntenin controls migration, growth, proliferation, and cell cycle progression in cancer cells. *Front Pharmacol.* 2015;6:241. 10.3389/fphar.2015.00241 26539120 PMC4612656

[42] Escola JM Kleijmeer MJ Stoorvogel W: Selective enrichment of tetraspan proteins on the internal vesicles of multivesicular endosomes and on exosomes secreted by human B-lymphocytes. *J Biol Chem.* 1998;273(32):20121–7. 10.1074/jbc.273.32.20121 9685355

[43] Kegelman TP Das SK Emdad L: Targeting tumor invasion: the roles of MDA-9/Syntenin. *Expert Opin Ther Targets.* 2015;19(1):97–112. 10.1517/14728222.2014.959495 25219541 PMC4632993

[44] Thompson CA Purushothaman A Ramani VC: Heparanase regulates secretion, composition, and function of tumor cell-derived exosomes. *J Biol Chem.* 2013;288(14):10093–9. 10.1074/jbc.C112.444562 23430739 PMC3617250

[45] Roucourt B Meeussen S Bao J: Heparanase activates the syndecan-syntenin-ALIX exosome pathway. *Cell Res.* 2015;25(4):412–28. 10.1038/cr.2015.29 25732677 PMC4387558

[46] Purushothaman A Bandari SK Liu J: Fibronectin on the Surface of Myeloma Cell-derived Exosomes Mediates Exosome-Cell Interactions. *J Biol Chem.* 2016;291(4):1652–63. 10.1074/jbc.M115.686295 26601950 PMC4722448

[47] Christianson HC Svensson KJ van Kuppevelt TH: Cancer cell exosomes depend on cell-surface heparan sulfate proteoglycans for their internalization and functional activity. *Proc Natl Acad Sci U S A.* 2013;110(43):17380–5. 10.1073/pnas.1304266110 24101524 PMC3808637

[48] Melo SA Luecke LB Kahlert C: Glypican-1 identifies cancer exosomes and detects early pancreatic cancer. *Nature.* 2015;523(7559):177–82. 10.1038/nature14581 26106858 PMC4825698

[49] Greene DK Tumova S Couchman JR: Syndecan-4 associates with alpha-actinin. *J Biol Chem.* 2003;278(9):7617–23. 10.1074/jbc.M207123200 12493766

[50] Choi Y Kim S Lee J: The oligomeric status of syndecan-4 regulates syndecan-4 interaction with alpha-actinin. *Eur J Cell Biol.* 2008;87(10):807–15. 10.1016/j.ejcb.2008.04.005 18621433

[51] Okina E Grossi A Gopal S: Alpha-actinin interactions with syndecan-4 are integral to fibroblast-matrix adhesion and regulate cytoskeletal architecture. *Int J Biochem Cell Biol.* 2012;44(12):2161–74. 10.1016/j.biocel.2012.08.017 22940199

[52] Dovas A Yoneda A Couchman JR: PKCbeta-dependent activation of RhoA by syndecan-4 during focal adhesion formation. *J Cell Sci.* 2006;119(Pt 13):2837–46. 10.1242/jcs.03020 16787950

[53] Bass MD Roach KA Morgan MR: Syndecan-4-dependent Rac1 regulation determines directional migration in response to the extracellular matrix. *J Cell Biol.* 2007;177(3):527–38. 10.1083/jcb.200610076 17485492 PMC1885470

[54] Hall A: Rho family GTPases. *Biochem Soc Trans.* 2012;40(6):1378–82. 10.1042/BST20120103 23176484

[55] Li H Peyrollier K Kilic G: Rho GTPases and cancer. *Biofactors.* 2014;40(2):226–35. 10.1002/biof.1155 24375503

[56] Gopal S Bober A Whiteford JR: Heparan sulfate chain valency controls syndecan-4 function in cell adhesion. *J Biol Chem.* 2010;285(19):14247–58. 10.1074/jbc.M109.056945 20154082 PMC2863221

[57] Mostafavi-Pour Z Askari JA Parkinson SJ: Integrin-specific signaling pathways controlling focal adhesion formation and cell migration. *J Cell Biol.* 2003;161(1):155–67. 10.1083/jcb.200210176 12695503 PMC2172880

[58] Gopal S Søgaard P Multhaupt HA: Transmembrane proteoglycans control stretch-activated channels to set cytosolic calcium levels. *J Cell Biol.* 2015;210(7):1199–211. 10.1083/jcb.201501060 26391658 PMC4586746

[59] Reijmers RM Spaargaren M Pals ST: Heparan sulfate proteoglycans in the control of B cell development and the pathogenesis of multiple myeloma. *FEBS J.* 2013;280(10):2180–93. 10.1111/febs.12180 23419151

[60] Rapraeger AC: Synstatin: a selective inhibitor of the syndecan-1-coupled IGF1R-αvβ3 integrin complex in tumorigenesis and angiogenesis. *FEBS J.* 2013;280(10):2207–15. 10.1111/febs.12160 23375101 PMC3651771

[61] Ramani VC Purushothaman A Stewart MD: The heparanase/syndecan-1 axis in cancer: mechanisms and therapies. *FEBS J.* 2013;280(10):2294–306. 10.1111/febs.12168 23374281 PMC3651779

[62] Theocharis AD Skandalis SS Neill T: Insights into the key roles of proteoglycans in breast cancer biology and translational medicine. *Biochim Biophys Acta.* 2015;1855(2):276–300. 10.1016/j.bbcan.2015.03.006 25829250 PMC4433619

[63] Barbareschi M Maisonneuve P Aldovini D: High syndecan-1 expression in breast carcinoma is related to an aggressive phenotype and to poorer prognosis. *Cancer.* 2003;98(3):474–83. 10.1002/cncr.11515 12879463

[64] Leivonen M Lundin J Nordling S: Prognostic value of syndecan-1 expression in breast cancer. *Oncology.* 2004;67(1):11–8. 10.1159/000080280 15459490

[65] Lim HC Multhaupt HA Couchman JR: Cell surface heparan sulfate proteoglycans control adhesion and invasion of breast carcinoma cells. *Mol Cancer.* 2015;14:15. 10.1186/s12943-014-0279-8 25623282 PMC4326193

[66] Ryu HY Lee J Yang S: Syndecan-2 functions as a docking receptor for pro-matrix metalloproteinase-7 in human colon cancer cells. *J Biol Chem.* 2009;284(51):35692–701. 10.1074/jbc.M109.054254 19858218 PMC2791000

[67] Khotskaya YB Dai Y Ritchie JP: Syndecan-1 is required for robust growth, vascularization, and metastasis of myeloma tumors *in vivo*. *J Biol Chem.* 2009;284(38):26085–95. 10.1074/jbc.M109.018473 19596856 PMC2758008

[68] Ramani VC Sanderson RD: Chemotherapy stimulates syndecan-1 shedding: a potentially negative effect of treatment that may promote tumor relapse. *Matrix Biol.* 2014;35:215–22. 10.1016/j.matbio.2013.10.005 24145151 PMC4377822

[69] Filmus J Capurro M: Glypican-3: a marker and a therapeutic target in hepatocellular carcinoma. *FEBS J.* 2013;280(10):2471–6. 10.1111/febs.12126 23305321

[70] Capurro M Martin T Shi W: Glypican-3 binds to Frizzled and plays a direct role in the stimulation of canonical Wnt signaling. *J Cell Sci.* 2014;127(Pt 7):1565–75. 10.1242/jcs.140871 24496449

[71] Haruyama Y Kataoka H: Glypican-3 is a prognostic factor and an immunotherapeutic target in hepatocellular carcinoma. *World J Gastroenterol.* 2016;22(1):275–83. 10.3748/wjg.v22.i1.275 26755876 PMC4698492

[72] Capurro M Wanless IR Sherman M: Glypican-3: a novel serum and histochemical marker for hepatocellular carcinoma. *Gastroenterology.* 2003;125(1):89–97. 10.1016/S0016-5085(03)00689-9 12851874

[73] Iglesias BV Centeno G Pascuccelli H: Expression pattern of glypican-3 ( *GPC3*) during human embryonic and fetal development. *Histol Histopathol.* 2008;23(11):1333–40. 18785116 10.14670/HH-23.1333

[74] Yamauchi N Watanabe A Hishinuma M: The glypican 3 oncofetal protein is a promising diagnostic marker for hepatocellular carcinoma. *Mod Pathol.* 2005;18(12):1591–8. 10.1038/modpathol.3800436 15920546

[75] Pilia G Hughes-Benzie RM MacKenzie A: Mutations in *GPC3*, a glypican gene, cause the Simpson-Golabi-Behmel overgrowth syndrome. *Nat Genet.* 1996;12(3):241–7. 10.1038/ng0396-241 8589713

[76] Lai JP Oseini AM Moser CD: The oncogenic effect of sulfatase 2 in human hepatocellular carcinoma is mediated in part by glypican 3-dependent Wnt activation. *Hepatology.* 2010;52(5):1680–9. 10.1002/hep.23848 20725905 PMC2967616

[77] Zittermann SI Capurro MI Shi W: Soluble glypican 3 inhibits the growth of hepatocellular carcinoma *in vitro* and *in vivo*. *Int J Cancer.* 2010;126(6):1291–301. 10.1002/ijc.24941 19816934

[78] Gao W Kim H Ho M: Human Monoclonal Antibody Targeting the Heparan Sulfate Chains of Glypican-3 Inhibits HGF-Mediated Migration and Motility of Hepatocellular Carcinoma Cells. *PLoS One.* 2015;10(9):e0137664. 10.1371/journal.pone.0137664 26332121 PMC4557904

[79] Venning FA Wullkopf L Erler JT: Targeting ECM Disrupts Cancer Progression. *Front Oncol.* 2015;5:224. 10.3389/fonc.2015.00224 26539408 PMC4611145

[80] Stewart MD Ramani VC Sanderson RD: Shed syndecan-1 translocates to the nucleus of cells delivering growth factors and inhibiting histone acetylation: a novel mechanism of tumor-host cross-talk. *J Biol Chem.* 2015;290(2):941–9. 10.1074/jbc.M114.608455 25404732 PMC4294521

[81] Oikari LE Okolicsanyi RK Qin A: Cell surface heparan sulfate proteoglycans as novel markers of human neural stem cell fate determination. *Stem Cell Res.* 2016;16(1):92–104. 10.1016/j.scr.2015.12.011 26722758

[82] Armulik A Genové G Betsholtz C: Pericytes: developmental, physiological, and pathological perspectives, problems, and promises. *Dev Cell.* 2011;21(2):193–215. 10.1016/j.devcel.2011.07.001 21839917

[83] Beard RE Zheng Z Lagisetty KH: Multiple chimeric antigen receptors successfully target chondroitin sulfate proteoglycan 4 in several different cancer histologies and cancer stem cells. *J Immunother Cancer.* 2014;2:25. 10.1186/2051-1426-2-25 25197555 PMC4155770

[84] Campoli M Ferrone S Wang X: Functional and clinical relevance of chondroitin sulfate proteoglycan 4. *Adv Cancer Res.* 2010;109:73–121. 10.1016/B978-0-12-380890-5.00003-X 21070915

